# Reliability of the Behaviorally Anchored Rating Scale (BARS) for assessing non-technical skills of medical students in simulated scenarios

**DOI:** 10.1080/10872981.2022.2070940

**Published:** 2022-05-04

**Authors:** Jaycelyn R Holland, Donald H Arnold, Holly R Hanson, Barbara J Solomon, Nicholas E Jones, Tucker W Anderson, Wu Gong, Christopher J Lindsell, Travis W Crook, Daisy A Ciener

**Affiliations:** a Division of Pediatric Emergency Medicine, Monroe Carell Jr. Children’s Hospital at Vanderbilt University Medical Center, Nashville, TN, USA; b Department of Biostatistics, Vanderbilt University Medical Center, Nashville, TN, USA; c Division of Pediatric Hospital Medicine, Monroe Carell Jr. Children’s Hospital at Vanderbilt University Medical Center, Nashville, TN, USA

**Keywords:** Behaviorally Anchored Rating Scale, crisis resource management, medical student education, non-technical skills, simulation

## Abstract

**Purpose:**

Caring for critically ill patients requires non-technical skills such as teamwork, communication, and task management. The Behaviorally Anchored Rating Scale (BARS) is a brief tool used to assess non-technical skills. The investigators determined inter- and intra-rater reliability of the BARS when used to assess medical students in simulated scenarios.

**Method:**

The investigators created simulation scenarios for medical students during their pediatric clerkship. Content experts reviewed video recordings of the simulations and assigned BARS scores for four performance components (Situational Awareness, Decision-Making, Communication, and Teamwork) for the leader and for the team as a whole. Krippendorff’s alpha with ordinal difference was calculated to measure inter- and intra-rater reliability.

**Results:**

Thirty medical students had recordings available for review. Inter- and intra-rater reliability for performance components were, respectively, Individual Situational Awareness (0.488, 0.638), Individual Decision-Making (0.529, 0.691), Individual Communication (0.347, 0.473), Individual Teamwork (0.414, 0.466), Team Situational Awareness (0.450, 0.593), Team Decision Making (0.423, 0.703), Team Communication (0.256, 0.517), and Team Teamwork (0.415, 0.490).

**Conclusions:**

The BARS demonstrated limited reliability when assessing medical students during their pediatric clerkship. Given the unique needs of this population, a modified or new objective scoring system for assessing non-technical skills may be needed for medical students.

## Introduction

Caring for a critically ill patient requires specific medical knowledge and technical skills related to resuscitation algorithms. However, in order for these skills to be implemented effectively, non-technical skills (NTS) are essential. In fact, the majority of errors in high-risk settings have been found to be related to deficiencies in NTS, as opposed to medical knowledge or technical skills [1,2]. Non-technical skills can be categorized to include cognitive or mental skills (such as decision-making, planning, and situational awareness), as well as social or interpersonal skills (team-working, communication, and leadership) [1]. While technical skills have been a focus of traditional medical training, the importance of NTS has become increasingly apparent. The publication of the report ‘To Err is Human’ by the Institute of Medicine emphasized the influence of human factors in the occurrence of medical errors and the need to address these in medical education [3]. Due to these findings, there has been a shift in medical training from focusing solely on medical knowledge to including behavioral aspects with NTS training [4].

### Background

Simulation and didactic-based curricula have been shown to be effective at improving NTS components in physicians at the resident and attending level in multiple clinical areas, including pediatrics, internal medicine, anesthesia, obstetrics, neonatology, surgery, and trauma. These studies do have limitations related to sample size as well as generalizability to all NTS or to other clinical settings [5–9]. There are also conflicting studies that show no improvement in NTS after didactic instruction when assessed in simulated scenarios [10] or that no additional improvement is seen after a single simulation session [11]. Ideally, improvements in NTS would be shown to have a similar impact in improving patient and safety outcomes. Literature regarding this concept is promising but limited [12]. Many studies focus on showing improvements in performance during simulated scenarios, such as the correlation of superior NTS with improved technical skills and adherence to national resuscitation protocols in simulated cardiopulmonary resuscitation [13] or faster time to resolution in simulated operating room crises [14]. The most promising intervention was a hospital-wide didactic program for medical team training, which was associated with lower surgical mortality [15]. These encouraging results highlight the importance and potential benefits of optimized NTS training as well as point out gaps in our research to this point in time.

### Medical student education and NTS

There is a specific lack of research to support the best method for integrating NTS in medical education [16]. The concept of introducing NTS training to medical students has evolved over the last two decades. Many studies focus on a specific component of NTS training, with communication skills being the most common [17,18]. Medical students have self-reported improvement in NTS skills after simulation training [19]. Studies showing improved clinical performance of technical skills after NTS training [20,21] are promising for the future of this field. However, there is considerable heterogeneity as to what outcomes are measured and there is little guidance available regarding the evaluation of NTS training with medical students. The primary limitation is a lack of a widely accepted scoring system for NTS in medical students [17].

An objective scoring system for NTS in medical students would allow for evaluation of curricula intended to improve NTS skills in this population. This is challenging as there are no generally accepted standardized measurements for NTS in most medical fields. Some specialties have developed their own measurement tools with satisfactory reliability and validity, such as the Anesthetists’ Non-Technical Skills (ANTS) [22] and the Non-Technical Skills for Surgeons (NOTSS) [23]. The ANTS tool is specifically developed for experienced anesthesiologists and typically requires a two-day training. There is concern that this would not translate well to medical students given the complexity of the training and the target population of attending anesthesiologists, limiting its practical application in undergraduate medication education.

The Behaviorally Anchored Rating Scale (BARS) (Appendix 1) was selected as a potentially appropriate test scale to assess NTS in the medical student population. It was originally adapted by anesthesiologists as a simpler alternative to the ANTS scale. The NTS domains assessed by BARS include situational awareness, decision-making, communication, and teamwork. The BARS showed promise with good inter- and intra-reliability when compared to the ANTS while only requiring 2 hours of training. This good inter- and intra-rater reliability persisted even when using third-year medical students to rate anesthesiology residents and student nurse anesthetists [24]. Given the reported simplicity of this tool as well as good performance when used by medical students as raters, it shows promise for application in evaluating undergraduate medical students. However, the BARS has not been used to assess medical students as a provider in a simulated scenario. The purpose of this study was to examine the inter- and intra-rater reliability of the BARS for NTS in medical students learning to manage pediatric emergencies.

## Methods

### Study design

This observational study involved a video review of clerkship-year medical student teams completing a simulation during their 2019–2020 core pediatric clerkship. Thirty students were included in the study. Each student rotated as the team leader once for a team of 2–3 other medical students participating in the study. We developed simulation scenarios in a high-fidelity simulation center. This center is located at a medical school within an urban, academic medical center. Simulations utilized an age-appropriate pediatric simulator (PediaSim, CAE Healthcare, Sarasota, FL). Scenario topics were selected from a consensus pediatric emergency medicine clerkship curriculum [25] and included asthma, anaphylaxis, croup, febrile seizure, and hypoglycemia. Students were provided with materials from the consensus curriculum prior to the simulation for prompting and self-directed preparation. Prior to the simulation, students received a standardized orientation to the simulation equipment. They were also asked to complete a survey with demographic information. A nurse confederate participated in the scenarios with a standardized script for each scenario topic. We recorded each scenario with a camera for review by raters. The authors’ Institutional Review Board determined this study to be exempt from oversight of human subjects research as it posed minimal risk to participants. Students were given the option to participate in the simulation sessions but were not included in the study for video review. We planned to complete simulation scenarios in the first, third, and fifth blocks of a five-block academic year as a convenience sample spread throughout the academic year. Due to the novel coronavirus pandemic, block five was cancelled. Using the BARS, the NTS domains of situational awareness, decision-making, communication, and teamwork were scored on a scale of one to nine. Scores were assigned both to the team leader and the team as a whole.

### BARS training

Prior to video review, content experts who would serve as raters of student NTS were provided with a two-hour training session on use of the BARS based on previously reported training by Watkins et al. Raters were provided materials on the BARS scale and literature to review prior to the training session. During the session, they were provided with instruction on the use of BARS and a matrix with specific examples of behavior in each of the BARS categories as utilized in the original BARS study (Appendix 2) [24]. After introductory training, the raters viewed a single video and formed a consensus rating of the student and team using the BARS rubric as a basis for their decisions. After consensus was obtained, the raters each viewed three additional videos and independently rated them. The videos for training were selected across a spectrum of performance from poor to satisfactory. The trainer had confidential discussions with each rater about the scores and provided feedback based on the rubric. Throughout this training process, practice scores obtained by raters were compared to consensus scores obtained by a prior panel of content experts. Data from training were not included in the analysis.

All content experts were pediatric emergency medicine (PEM) faculty who had completed a pediatrics or internal medicine/pediatrics residency, a pediatric emergency medicine fellowship, and had obtained faculty appointment at the tertiary care academic institute. They had varying years of experience that were similar between the consensus group and the raters. Neither the consensus group nor the raters had any interactions with the students before or during the study. The first author facilitated the simulation scenarios to assist with data capture and performed the video review training. The raters were not provided any identifiable information about the students other than what they were able to view in the videos.

### Video review

To generate the study data, video review occurred during two separate sessions, 1 week apart. During each session, all videos were presented once, with the order randomized separately for each session. After each video, raters independently scored the student and the team using the BARS. Scores were recorded and managed using REDCap (Research Electronic Data Capture) [26]. Three raters scored all students’ performance videos twice, 1 week apart, on a 1–9 ordinal scale.

### Statistical analysis

Inter-rater agreement was calculated across the three raters, and intra-rater agreement was calculated between the first and second ratings. The study used Krippendorff’s alpha as the reliability measurement [27]. Krippendorff’s alpha is applicable to more than two raters (the study had three) and accounts for the ordinal rating scale. Krippendorff’s alpha is a chance-corrected agreement coefficient and takes values from 0 to 1 where zero means no agreement, accounting for chance agreement. In interpreting alpha, it is commonly accepted that a value of 0.8 or greater reflects excellent agreement. For scales with an alpha below about 0.667, reliability may be insufficient to support conclusions based on the scale as an outcome measure [28]. All the analysis was conducted in R using package ‘irr’ (R Foundation for Statistical Computing, Vienna, Austria).

## Results

Thirty students participated in simulation scenarios as a team leader and had videos available for review. All eligible students elected to participate in the study. Demographic information on participating students is shown in Table 1. Of the 30 students, 21 (70%) had not witnessed a code or resuscitation prior to participation in the simulation curriculum. Seven (23.3%) had medical experience prior to medical school, including emergency medical technicians (EMTs), nurses, nurse aids, and cardiopulmonary resuscitation (CPR) instructors.

**Table 1.** Demographic characteristics of 30 second-year medical students participating in pediatric emergency simulation curriculum

Characteristic	
Age, years: mean (SD)	24.4 (1.6)
Gender, %	
Female	56.7
Male	43.3
Race, %	
White	60.0
Asian	16.7
Black	3.3
White and Asian	10.0
White and Hispanic	3.3
Other	6.7
Intended Career Field, %	
Anesthesiology	3.3
Emergency Medicine	3.3
Internal Medicine	13.3
Obstetrics and Gynecology	3.3
Pediatrics	16.7
Surgery	10.0
Undecided	50.0
Witnessed a code or resuscitation, %	
Yes	30.0
No	70.0
Training prior to medical school, %	
EMT	13.3
EMT and CPR Instructor	3.3
Nurse	3.3
Nurse Aid	3.3
PhD	3.3
None	73.3

Abbreviations: SD, Standard Deviation; EMT, Emergency Medical Technician; CPR, Cardiopulmonary Resuscitation; PhD, Doctor of Philosophy

Assigned scores are summarized in Table 2. Mean scores for each component of BARS ranged from 2.4 to 2.7. All scores for each component of BARS ranged between 1 and 7, with no students or teams assigned scores of 8 or 9 on any of the components. Inter-rater and intra-rater reliability for each BARS component are shown in Table 3. Generally, intra-rater reliability exceeds inter-rater reliability, but neither achieves excellent reliability for individual or team scores across any of the domains. While the intra-rater reliability might be considered moderate, the inter-rater reliability exceeded 0.5 for only one measure.

**Table 2.** Summary of rated items on a scale from 1 to 9 on the Behaviorally Anchored Rating Scale (BARS) evaluating second-year medical students in simulated pediatric emergencies

BARS Domain	Mean (SD)	Median [IQR]	Median [Range]
Individual Situational Awareness	2.5 (1.2)	2.0 [2.0, 3.0]	2.0 [1.0, 7.0]
Individual Decision Making	2.4 (1.2)	2.0 [1.0, 3.0]	2.0 [1.0, 7.0]
Individual Communication	2.5 (1.2)	2.0 [2.0, 3.0]	2.0 [1.0, 7.0]
Individual Teamwork	2.4 (1.1)	2.0 [2.0, 3.0]	2.0 [1.0, 7.0]
Team Situational Awareness	2.7 (1.2)	3.0 [2.0, 3.0]	3.0 [1.0, 7.0]
Team Decision Making	2.6 (1.2)	2.0 [2.0, 3.0]	2.0 [1.0, 7.0]
Team Communication	2.7 (1.1)	3.0 [2.0, 3.0]	3.0 [1.0, 7.0]
Team Teamwork	2.6 (1.1)	2.0 [2.0, 3.0]	2.0 [1.0, 7.0]

Abbreviations: SD, Standard Deviation; IQR, Interquartile Range

**Table 3.** Inter- and intra-rater reliability of the Behaviorally Anchored Rating Scale (BARS) evaluating second-year medical students in simulated pediatric emergencies

BARS Domain	Inter-Rater Reliability (Krippendorff’s Alpha)	Intra-Rater Reliability (Krippendorff’s Alpha)
Individual Situational Awareness	0.488	0.638
Individual Decision Making	0.529	0.691
Individual Communication	0.347	0.473
Individual Teamwork	0.414	0.466
Team Situational Awareness	0.450	0.593
Team Decision Making	0.423	0.703
Team Communication	0.256	0.517
Team Teamwork	0.415	0.490

## Discussion

Our study indicates that the intra- and inter-rater reliability of the BARS may be insufficient to support its routine use for evaluating medical student NTS performance during simulated pediatric emergencies. All medical students should graduate with a foundational understanding of NTS; this has been identified as a deficiency in medical school training. Without further development of an appropriate rating scale with acceptable validity and reliability, curricula designed to teach NTS will remain non-evidence based. Due to a lack of a widely adopted framework for training and evaluating NTS in health care existing tools are generally locally developed and not validated for the medical student population [29,30]. A scale with performance similar to or superior to that of the ANTS [22] or NOTSS [23] would advance our understanding of training medical students in NTS. Our data suggest that BARS does not meet this need. One newer possibility is the Anaesthesiology Students’ Non-Technical Skills (AS-NTS) scale, which was developed and published in 2019 outside of Germany. This examined 2nd through 4th year undergraduate medical students in emergency medicine and anesthesiology scenarios. It showed good interrater reliability of its components as well as content validity [31]. This tool has since been used to investigate the relationship of student situational motivation with NTS performance [32] as well as the influence of flipped learning interventions to improve NTS performance [33]. Per the authors, the initial study was performed in German language at one institution with a limited number of teachers. Further studies could be conducted regarding the English-language version to further establish validity, reliability, and feasibility in multiple educational applications.

We chose to evaluate the BARS due to a shorter time required for training as well as prior reliability demonstrated with medical student raters [24]. Our scores showed a wide range, with scores between 1 and 7, though the mean scores ranged from 2.4 to 2.7. The authors have several theories regarding these findings. Given that medical students have limited clinical experience at this time in their training, it may be challenging to assess NTS in the setting of limited medical knowledge to care for the simulated patients. Also, the short training time of 2 hours may not have been adequate for the raters to gain familiarity with the scale. However, this amount of training was sufficient in the Watkins study and, from a usability standpoint, it would be challenging to adopt a scale into usual practice that required more training.

To our knowledge, this is one of the earliest experiences formally evaluating NTS within the medical school curricula, and one of the few studies utilizing an objective scale as opposed to checklists or self-reported response from students. The ideal timing for beginning NTS education for physicians is unknown. Residents and interns report feeling unprepared for the assessment and initial management of acutely ill patients, both regarding technical and non-technical skills [34,35], though they will likely be the first provider called to the bedside. Earlier in a physician’s training, medical education in critical care (including technical and non-technical skills) is largely elective, scattered, and highly variable across the country [36]. There has been recent development of training programs to improve medical students’ skills in managing acutely decompensating patients. However, these programs have typically focused on technical skills. While they do not include NTS training, they do show the promise of using simulation to train medical students in critical care [37–39]. As medical students have reported avoiding resuscitations due to a lack of training [40], introduction of simulated emergencies early into the medical school curriculum could improve student exposure to critical learning opportunities.

### Limitations

Our study has limitations. The number of students, and thus simulations, was partially limited due to cancellations necessary during the coronavirus pandemic. This was many of these students’ first experience with simulation of emergent situations, and it is unclear if inter- or intra-rater reliability would differ for more experienced trainees who perform above ‘poor’ on the BARS. Lack of familiarity with the simulation equipment and environment may have affected student technical skills with consequent impact on NTS. The scenarios were chosen from topics in a pediatric emergency medicine consensus curriculum to be appropriate for the students’ level of training. These scenarios were of lower acuity than the scenarios described in Watkins et al. for the initial study of the BARS. It is unknown if the assessment of NTS is impacted by the complexity of the technical skills required to complete the tasks at hand. Our training was brief, based on prior training reported by Watkins et al. – future studies could investigate the impact of a more in-depth training or a larger number of student participants on reliability.

## Conclusions

While the BARS did not show good reliability, our result are broadly informative in the field of medical education. We show that directly applying existing assessment tools to medical students may not be appropriate and that new or modified assessment tools will be needed for evaluating NTS among this learner group. While we do not expect modifications to the simulation training to improve the inter- and intra-rater reliability of the BARS, more extensive training on the BARS and assessing the evaluators’ learning curve could expose the benefits of this tool not supported by the current data. Once a reliable tool is identified, educators can begin to study the optimal approaches for training medical students in NTS. Given the importance of NTS for practicing physicians and the potential implications on patient outcomes, it is imperative that we obtain a reliable assessment tool for assessing NTS in undergraduate medical education.

## Appendix 1. Behaviorally Anchored Rating Scale (BARS) for Evaluating Non-Technical Skills of Medical Students in Simulated Pediatric Emergencies

**Table:**

Leader	Situational Awareness									Decision Making									Communication									Teamwork								
	Poor			Medium			Excellent			Poor			Medium			Excellent			Poor			Medium			Excellent			Poor			Medium			Excellent		
	1	2	3	4	5	6	7	8	9	1	2	3	4	5	6	7	8	9	1	2	3	4	5	6	7	8	9	1	2	3	4	5	6	7	8	9
Team	Situational Awareness									Decision Making									Communication									Teamwork								
	Poor			Medium			Excellent			Poor			Medium			Excellent			Poor			Medium			Excellent			Poor			Medium			Excellent		
	1	2	3	4	5	6	7	8	9	1	2	3	4	5	6	7	8	9	1	2	3	4	5	6	7	8	9	1	2	3	4	5	6	7	8	9

## Appendix 2. Behaviorally Anchored Rating Scale (BARS) Matrix

[Originally published in 24]
